# Utilizing Water Activity as a Simple Measure to Understand Hydrophobicity in Ionic Liquids

**DOI:** 10.3389/fchem.2019.00112

**Published:** 2019-03-05

**Authors:** Qi Han, Xungai Wang, Nolene Bynre

**Affiliations:** Institute for Frontier Materials, Deakin University, Geelong, VIC, Australia

**Keywords:** ionic liquid, hydrated IL, hydrogen bond basicity, water activity, NMR, hydrophobicity

## Abstract

Ionic liquids (ILs) are regarded as designable solvents finding use in a variety of applications. One of the challenges of the design and selection process is to understand the ionic liquid properties. In this work, we selected seven ILs containing three types of hydrophilic anions and examined several key properties, which are correlated to hydrophobicity. In particular, we measured the hydrogen bond basicity β and water activity a_w_ of IL and IL-water mixtures, and suggested that these two properties are linearly correlated particularly in hydrated ILs. We then used NMR to evaluate the chemical shift of H_2_O in hydrated ILs. Correlating the outcomes of each of these techniques with respect to understanding the hydrophobicity of the ILs is discussed. It is shown that water activity a_w_ is the most facile technique to represent and understand hydrophobicity of ILs.

## Introduction

Ionic liquids (ILs) are salts with melting points below 100°C. One of the most attractive properties of ILs is the tunability of the component ions, and extensive efforts have been devoted to understanding the properties of various ILs (Welton, 1999; Forsyth et al., 2004; Plechkova and Seddon, 2008; Freemantle, 2010; Hallett and Welton, 2011). Some key IL properties include polarity, hydrophobicity, viscosity, purity, and Kamlet Taft parameters (Olivier-Bourbigou et al., 2010). However, some of the important properties of ILs such as hydrophobicity cannot be easily measured, while a few of these properties may be correlated but require more studies.

Hydrophobicity of ILs is considered in a wide range of applications. It has been demonstrated that the IL hydrophobicity influences its solvation ability, reaction rates, reaction mechanisms, product yields, and enzyme activity, etc. (van Rantwijk and Sheldon, 2007; Hallett and Welton, 2011). While water is the most universal solvent and considered as impurity or co-solvent of ILs (Li et al., 2010, 2012; Patel et al., 2014; Han et al., 2017), hydrophobicity represents the miscibility with water. However, there is no normalized scale for the hydrophobicity. Generally, the Log P scale has been used to quantify the hydrophobicity of ILs, which is defined as the logarithm of their partition coefficient P of un-ionized ILs between octanol and water. Whereas in some cases, an IL partitions in an octanol/water mixture as an ion pair, and hence the log P value may depend not only on the concentration of the cation but also on that of the anion, and of ion pairing in both phases (Kaar et al., 2003; Yang and Pan, 2005). Conversely, a number of researchers predicted IL Log P by computation (Chapeaux et al., 2007; Mutelet et al., 2011). Thus, Log P can hardly be measured and present the hydrophobicity of ILs, but it can be a useful value for the prediction of the hydrophobicity.

The hydrophobicity can be regarded as a subset concept of polarity. The polarity is associated to the solubility of substrates/products and water association between solvent and solute (Zaks and Klibanov, 1988). The polarity of ILs can be quantified as Kamlet-Taft (KT) solvatochromic parameters, which is based on the analysis of the UV-Vis spectral band shifts of solvatochromic probes (Kamlet et al., 1981) (Oehlke et al., 2006). The KT parameters include hydrogen bond acidity α, hydrogen bond basicity β and dipolarity/polarizability π^*^, while β is considered as a significant parameter, since it specifically describes the solvent ability to donate electron density to form a hydrogen bond with protons of a solute (Ab Rani et al., 2011). Thus, β is related to the tendency of ILs to form hydrogen bonds with the water molecule and can be considered as an important indicator of hydrophobicity of ILs. Meanwhile, the nucleophilicity is related to polarity and particularly β (Zhao, 2016), but it cannot be measured and will be incorporated in the concept of polarity. KT parameters are measurable, however, the major drawback of their measurement is that the measurement depends on the set of probe dyes used, and it is sensitive to measurement conditions, such as the color and purity levels of the ILs and procedures employed. Recently, numerous studies utilized water as a co-solvent of ILs to modulate β and thus hydrogen bonding interaction, e.g., hydrated ILs by adding a small amount of water (~75 mol%) (Ohno et al., 2015) and IL-water mixtures (~90 mol%) (Lai et al., 2011; Han et al., 2016).

From the aspect of water, the contribution of water is associated with the free water rather than the water content added in the solvent (Zaks and Klibanov, 1988). And this can be quantified by the thermodynamic water activity (*a*_w_). *a*_w_ is defined as the ratio of the partial pressure of aqueous salt solutions (*p*) to that of pure water (*p*0) (*i.e., a*_w_ = *p*/*p*0) (Cauvain and Young, 2009). Nowadays, *a*_w_ can be measured using certain instruments (Ohno et al., 2015). Besides, another property of ILs, kosmotropicity, describes an ion's ability to facilitate the structuring of nearby water molecules. Previous studies have suggested that the hydration state of an IL correlates with its stabilization effect on the dissolved proteins (Fujita et al., 2007). And recent studies have shown that the chemical shift of H_2_O in ILs using nuclear magnetic resonance is related to the state of the hydrogen bonding network (Sare et al., 1973; Saihara et al., 2015) and even the kosmotropicity (Nikawa et al., 2017).

**Figure 1:**
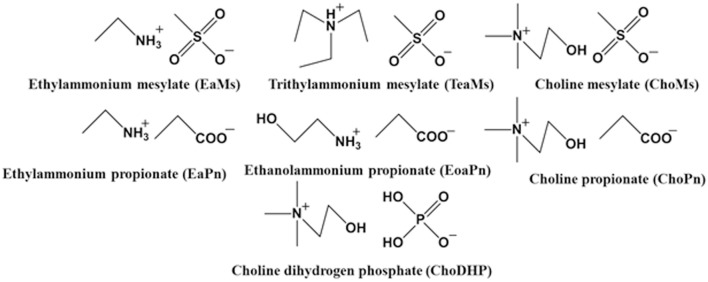
Structure of ionic liquids used in this study.

It has been demonstrated that the chemical structure of the ILs such as the chain length of cation and unique structure of anion [e.g., bis(trifluoromethylsulfonyl)imide (Tf_2_N^−^) and hexafluorophosphate (${\text{PF}}_{6}^{-}$)] influences the hydrophobicity of ILs (Cammarata et al., 2001). Since most ILs tend to be hydrophilic (water-miscible), to investigate the rule of their hydrophobicity was the focus of this work. In the present work, we selected seven simple ILs to understand the correlation between measured properties and hydrophobicity of ILs. The ammonium cations coupled with hydrophilic anions including propionate, mesylate and dihydrogen phosphate were selected (Figure 1). The hydrogen bond basicity β and water activity of the ILs and hydrated ILs were explored, and the chemical shift of H_2_O in hydrated ILs was subsequently measured. Then the Log P of the conjugated base and acid was evaluated. By correlating the results of these measurements, the hydrophobicity of the ILs is discussed.

## Methods

### Synthesis of ILs

All starting reagents were commercially obtained unless further mentioned, and used without further purifications. The ILs [ethylammonium mesylate (EaMs), triethylammonium mesylate (TeaMs), choline mesylate (ChoMs), ethylammonium propionate (EaPn), ethanolammonium propionate (EoaPn), choline propionate (ChoPn), choline dihydrogen phosphate (ChoDHP)] were synthesized according to the reported method (Belieres and Angell, 2007; Han et al., 2016). In brief, the ILs were synthesized by neutralizing equimolar amounts of the corresponding acid and base. A typical example of the procedure is as follows. Triethylamine (5.06 g, 0.05 mol) was added drop-wise to methanesulfonic acid (4.80 g, 0.05 mol) in a round bottom flask (250 mL) whilst stirring with a magnetic stir bar in an ice bath for 2 h. The mixture was allowed to stir overnight at room temperature and then dried under reduced pressure for 8 h at 40°C. After drying, a transparent clear liquid (9.85 g) was obtained. The water content of the synthesized ILs (<1 wt%) was measured by a Karl Fisher coulometer (Metrohm 899, MEP Instruments). The water content of EaMs, TeaMs, ChoMs, EaPn, EoaPn, ChoPn, ChoDHP was 0.50, 0.81, 0.89, 0.10, 0.93, 1.00, and 0.60 wt%, respectively. NMR samples of synthesized ILs were prepared in deuterated dimethyl sulfoxide (DMSO-d6). NMR experiments were acquired at 298 K (20°C) using 500 MHz Bruker Avance III nuclear magnetic resonance spectrometer (^1^H at 500.130 MHz) equipped with a 5 mm broad-band probe. Chemical shifts (δ) are expressed in ppm with reference to the residual solvent signal (2.500 ppm for DMSO).

### Characterization

The hydrogen bond basicity β of ILs and IL-water mixtures was measured and calculated as reported (Hauru et al., 2012; Debeljuh et al., 2013). Experimental conditions were slightly modified based on methanol and water, and the obtained values of methanol and water were in good agreement with the literature (Deye et al., 1990). First, a specified amount of ILs was gravimetrically mixed with MilliQ water to reach the required concentrations. The stock solutions of 4-nitroaniline (NA, 1 mol/L) and *N*,*N*-diethyl-4-nitroaniline (DENA, 1 mol/L) were dissolved in methanol prior to use. A 1 μL portion of the stock solution was transferred to 1 mL IL solutions (neat, 25 mol% or 8.3 mol% ILs) and the mixture was vigorously agitated using a vortex. The *λ*max of each sample was obtained from using UV/vis spectrophotometry. Deionized water or blank IL samples were measured and were background subtracted. UV/vis measurements for each sample were measured at room temperature and were repeated at least twice. The peak of the spectra was fitted with a Gaussian function in order to precisely locate the maxima (*λ*max). The result was a resolution exceeding that of the instrument (1 nm). The β was calculated according to equations 1 and 2,

(1) $$\nu max/1000\;cm{\;}^{-1}=\frac{1}{0.0001\lambda max/nm}$$

(2) $$\beta \;=\;\frac{1.035\;\nu max\left(DENA\right)+2.64-\nu max(NA)\;}{2.8}$$

where *λ*max (nm) is the maximum wavelength, while νmax (DENA) and νmax (NA) are the wavenumbers (1,000 cm^−1^) at maximum absorbance for 4-nitroaniline and *N*,*N*-diethyl-4-nitroaniline, respectively (Kamlet and Taft, 1976). The mean deviations for β were < ± 0.01. The water activity of 3 mL IL-water mixtures (at 8.3 mol%, 10 mol%, 12.5 mol%, 16.7 mol%, 25 mol%, 33.3 mol% or neat ILs) was measured using a water activity instrument (LabSwift-a_w_, Novasina AG). The solutions were placed in the container of the instrument and placed in the panel for readings. All the measurements were determined in a nitrogen atmosphere (Aldrich AtmosBag, Sigma-Aldrich) with triplicate readings (mean deviations < ± 0.03). The NMR experiments of the hydrated ILs were prepared in CDCl_3_ using a coaxial insert. The predicted Log P and ALogP of the ILs in this study was estimated Chemicalize (ChemAxon)^1^ and VCCLab (VCCLAB),^2^ respectively.

## Results and Discussion

### Hydrogen Bond Basicity β of the ILs

Since the hydrogen bond basicity β is possibly an important indicator for the evaluation of hydrophobicity, β of the seven ILs was initially evaluated. Table 1 shows β of the ILs including neat (~100% IL), hydrated (25 mol%) and IL-water mixtures (8.3 mol%). In particular, the last two concentrations have been identified as the boundary of different states of ILs. ILs at ~25 mol% presented as typical hydrated ILs where molar ratio of IL and H_2_O is 1:3 with no free water, while IL-water mixtures (~8.3 mol% ILs) contained incompletely dissociated ions (Zhang et al., 2008; Kohno and Ohno, 2012; Stange et al., 2013; Ohno et al., 2015; Han et al., 2017). As the some of the ILs were not room-temperature ILs, the β of neat ILs was not obtained. As reported, β is mainly controlled by the IL anion (Ab Rani et al., 2011), and hence the seven ILs were categorized as three groups based on the anions.

**Table 1:** β values of ILs and IL-water mixtures in this study.

**ILs**	**β of neat ILs**	**β of hydrated ILs (25 mol%)**	**β of ILs at 8.3 mol%**
EaMs	–	0.57	0.29
TeaMs	0.74	0.59	0.42
ChoMs	–	0.46	0.18
EaPn	1.02	0.64	0.40
EoaPn	0.91	0.63	0.36
ChoPn	0.98	0.70	0.57
ChoDHP	–	0.26	0.25

–, *unmeasurable due to the high melting point of the ILs*.

It is seen that the intrinsic β values of four neat ILs were up to 0.7, while the three propionate-based ILs had similar β (~1.0). β of ChoPn was slightly higher than other two ILs, whereas the difference among the three ILs was not distinguishable. Regarding hydrated ILs (25 mol%), the β values decreased along with the dilution effect on the ILs. β of propionate-based ILs was in the range of 0.63–0.7 and was higher than that of mesylate-based ILs (0.46–0.59), while ChoDHP showed the lowest β value (0.26). Another study has also reported the similar order in neat or concentrated ILs (Zhao, 2016). In IL-water mixtures (8.3 mol%), the β values further reduced, while the trend of β was observed as same as it in hydrated ILs, i.e., propionate-based ILs > mesylate-based ILs > ChoDHP. Notably, β of ChoPn still showed the highest value among the ILs, and this larger β value implies that choline has a greater tendency to accept protons from the probe dye when coupled with propionate than other combinations of cations and anions. β of mesylate-based ILs varied from 0.18 to 0.42, while ChoMs showed the lowest β in the seven ILs. Generally, the decreasing rate of β values as a function of IL concentration was different. For example, from 25 to 8.3 mol%, β of ChoMs dropped from 0.46 to 0.18, however, it was 0.26–0.25 for ChoDHP. This may be due to the incompletely dissociated ions of ILs in 8.3 mol% and such dissociation varies with different types of ILs (Zhang et al., 2008; Stange et al., 2013). In addition, it has been reported that β of aqueous ILs was not linear as a function of water, while the tendance was slightly different for ILs holding the same anion (Debeljuh et al., 2013). However, it observes that ChoDHP remained ~0.25 over the dilution, indicating that choline cation may become a weaker hydrogen bond acceptor when coupling with dihydrogen phosphate.

Considering that β refers to the solvent ability to form a hydrogen bond (Ab Rani et al., 2011), it can be implied that mesylate-based ILs with lower β have a lower tendency to form hydrogen bonds with water molecule, and hence they are less hydrophilic than propionate-based ILs, while ChoDHP has the lowest measured β values and therefore is likely to have less hydrogen bonding interactions. It can be proposed that this rule can also be applied to the evaluation of the affinity of ILs with water and hence hydrophobicity of ILs. It is noticeable that the anion dominates the β values, and propionate anion tends to be basic while mesylate anion tends to be neutral as reported (Pagni, 2003; MacFarlane et al., 2006).

### Water Activity as a Function of IL Concentration

**Figure 2:**
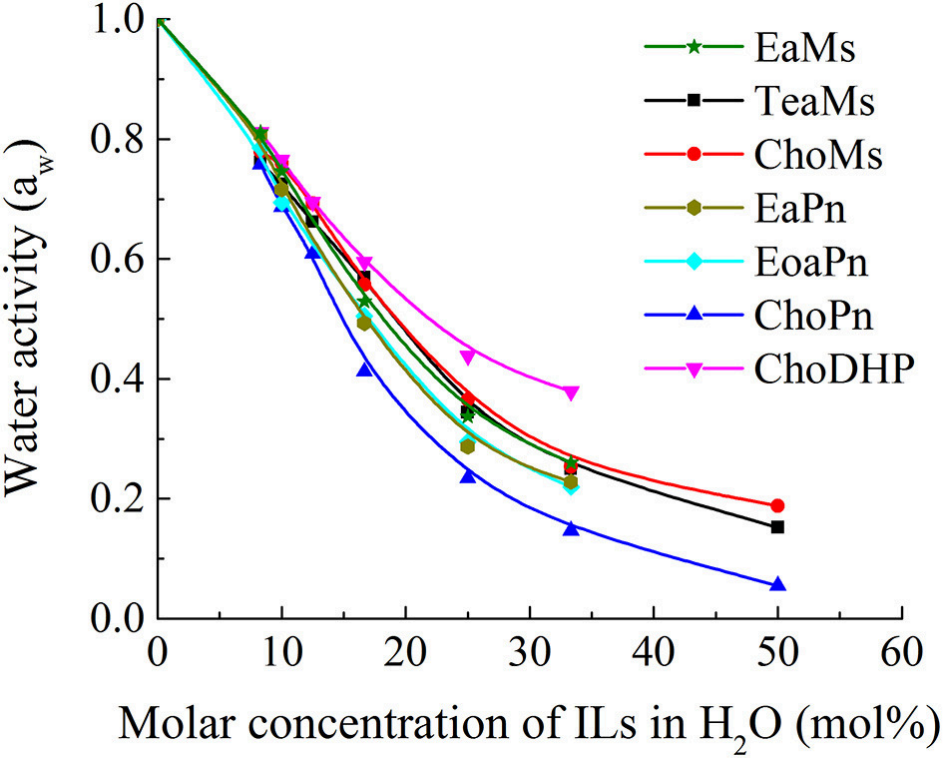
Water activity of ILs as a function of molar concentration.

The role of water in ionic liquids is important and many applications of IL use water as a co-solvent. Strong interactions of water molecules with the ions of ILs reduce the vapor pressure, which can be measured by water activity a_w_. Thus, the high values of a_w_ (up to 1) refers to low interaction/affinity of ILs with water, and vice versa. In Figure 2, it is observed that the water activity a_w_ decreased from 1 to almost 0 as a function of the molar concentration of ILs in the solution. At low IL concentrations (0–20 mol%), water activity a_w_ decreased rapidly, while the decreasing rate was relatively steady in IL solutions with more than 20 mol% ILs. This indicates that the interaction between water and ILs becomes stronger in the hydrated state (<25 mol%), while the decreasing rate of a_w_ was reduced probably owing to the weakened interaction between water and ILs and the partial dissociation of ions. Meanwhile, the differences of a_w_ among the seven ILs was more distinguishable within this range (around 25 mol% ILs). This range has been highlighted as the hydrated state of IL, which contains no free water and has been applied as biological systems (Ohno et al., 2015; Han et al., 2016). Thus, the comparison among the ILs was focused in this region, i.e., 25 mol%.

For the seven ILs particularly at 25 mol%, the overall trend for water activity as function of water concentration is very similar to β values measured above. The order of a_w_ shows ChoDHP > propionate-based ILs > mesylate-based ILs, suggesting that ChoDHP showed the lowest affinity with water, and ChoPn had the highest affinity. The three mesylate-based ILs showed very similar values across the entire water concentration range measured. The two ILs EaPn and EoaPn had similar trend along the IL concentrations, while they were slightly higher than ChoPn. It is known that ChoPn possesses strong hydrogen bonding capabilities (β = 0.98) and thus is the most hydrophilic among the seven ILs (Fukaya et al., 2007; Patinha et al., 2015). In addition, while TeaMs and ChoMs with chemically different cations shared a similar a_w_, this indicates that the anion mesylate significantly influenced the hydrophobicity. This is in agreement with previous studies, which showed that the anion plays an important role on the IL properties (MacFarlane et al., 2006; Sate et al., 2007), and is primarily involved in the formation of hydrogen bonding (Cammarata et al., 2001). However, for ILs holding the same anion, the hydrophobicity of ILs cannot be easily identified as a function of cation.

**Figure 3:**
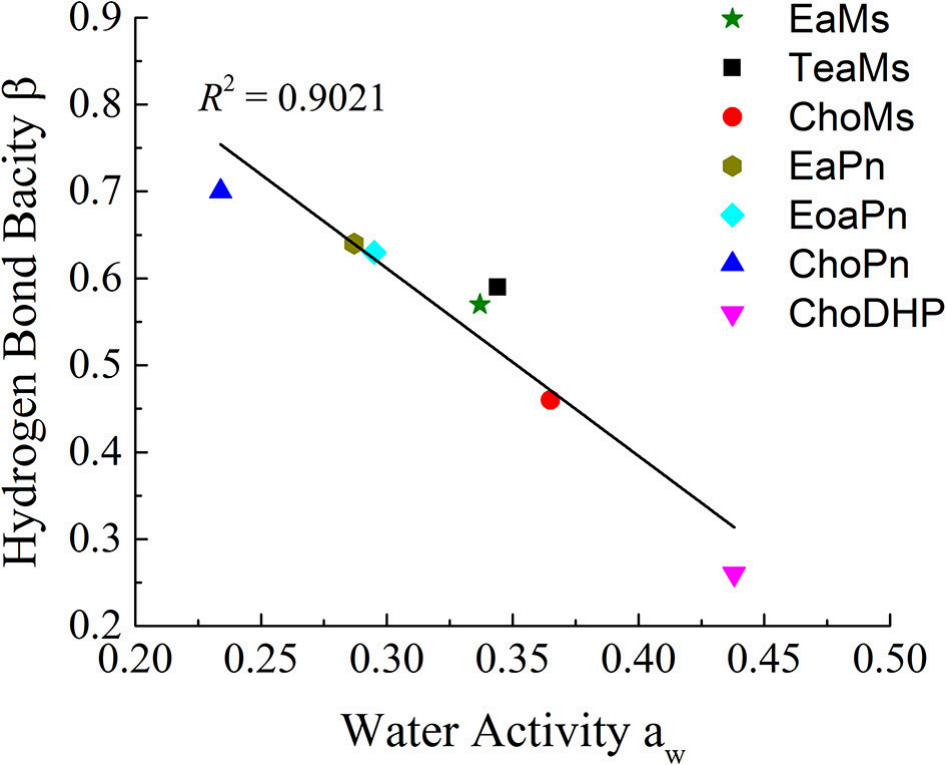
The linear correlation between hydrogen bond bacisity β and water activity a_w_ of hydrated ILs (25 mol%).

Since Figure 2 shows the clear trend of a_w_ in hydrated ILs (25 mol%), we correlated β with a_w_ at the same concentration of ILs (Figure 3). In particular, at 25 mol%, the molar ratio of IL and water is 1:3, which forms hydrated condition of ILs without free water. In this case, depending on the hydrophobicity, the properties of hydrated ILs such as β, a_w_ are impacted. Figure 3 demonstrated the inverse linear relationship between β with a_w_, where *R*^2^ reached 0.9021. It should be noted that in the seven points, there are no critical points i.e., extreme high beta with low a_w_ or extreme low beta with high a_w_, we suggest that this relationship is a linear relationship in such range. ChoPn showed the highest β up to 0.70 and the lowest a_w_ (0.234), while ChoDHP had the lowest β reaching 0.26 and highest a_w_ (0.438). The order of hydrophobicity can be described as ChoPn < EaPn ≈ EoaPn < EaMs ≈ TeaMs < ChoMs < ChoDHP. Again, the anion plays a key role on these two properties, however, the differences between EaPn and EoaPn, EaMs and TeaMs were relatively indistinctive. This may be because they shared the similar cations and same anions. Considering the nature of β and a_w_ as discussed above and their linear correlation, it can be suggested that the trend of water activity directly represents the different hydrogen bond network (expressed by β) in IL solutions, and hence hydrophobicity of ILs.

In general, both β and a_w_ of the seven ILs have a clear trend at 25 mol%, which is useful to understand the hydrophobicity of ILs. However, for a_w_ of diluted ILs (lower than 15 mol%) and β of neat ILs, the difference among the ILs was not significant (Table 1 and Figure 1). Taken the test method into consideration, the measurement of β has a few drawbacks, such as the sensitivity of ILs to the set of probe dyes used and to measurement conditions. The evaluation of a_w_ at 25 mol% ILs is likely to be a more facile approach to understand the hydrophobicity of ILs.

### Chemical Shift of H_2_O in Hydrated Ionic Liquids

**Figure 4:**
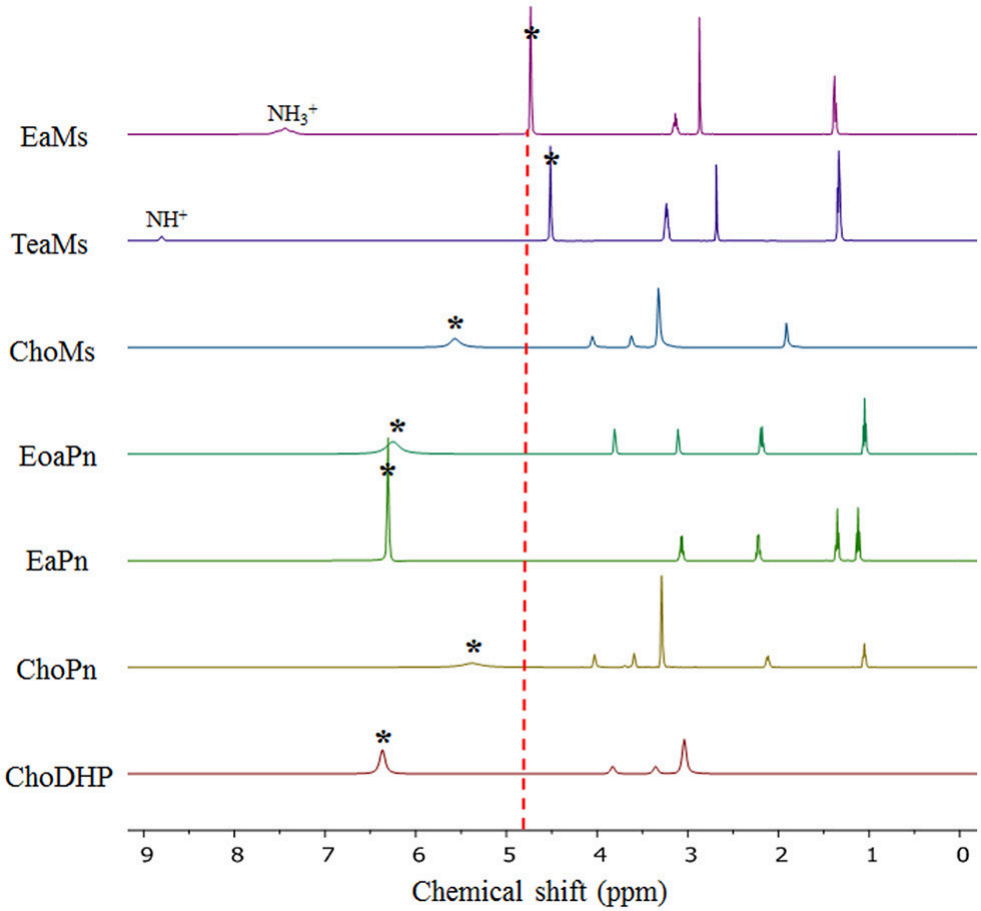
NMR diagram of the hydrated ILs (25 mol%) at 20°C. The asterisk (*) refers to the signal of H_2_O in the ILs. The NH peaks of protic ILs were marked. The dotted vertical line represents the signal of neat H_2_O (4.80 ppm).

Here, NMR is used to evaluate the formation of hydrogen bonding networks between ionic liquids and water molecules. The chemical shift of H_2_O in hydrated ILs can reveal hydration state of the ions, which also represented the kosmotropicity (Nikawa et al., 2017). In Figure 4, it is observed that the chemical shift of H_2_O in hydrated ILs (25 mol%) varied based on the chemical shift relative to that of neat water (4.81 ppm).

It is evident that the chemical shift of H_2_O in EaMs and TeaMs shifted slightly upfield to 4.73 and 4.50 ppm, respectively. As reported, the upfield shift suggests that the formation of hydrogen bonding network was promoted by the ILs (Nikawa et al., 2017). However, these two ILs are protic ILs with transferred protons, which may be not comparable with other aprotic ILs. Moreover, the upfield shift of NH peak was noted, suggesting the water may influence the proton transfer. Interestingly, the other two protic ILs, EoaPn and EaPn showed the water peak in downfield, and their NH peaks were merged into the signal of water. This may imply that water was fast exchanged with the transfer proton in EoaPn and EaPn, and hence the merge with NH peak led to the downfield shift of water peak. Therefore, these two ILs formed stronger hydrogen bonding with water than EaMs and TeaMs. This result is supported by the β and a_w_ curves of ILs (Table 1 and Figure 2). In other words, EoaPn and EaPn were more hydrophilic than EaMs and TeaMs.

**Figure 5:**
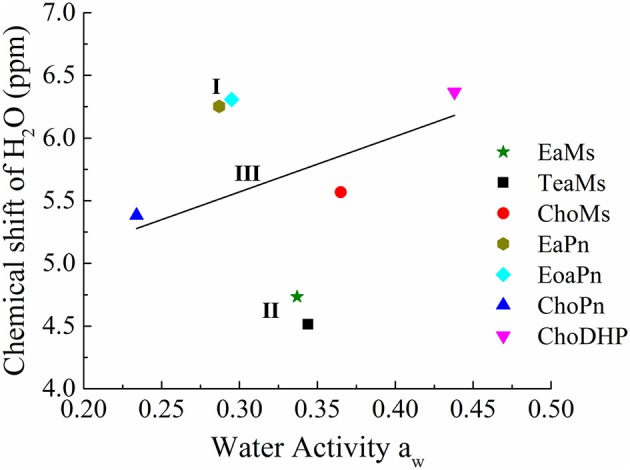
The correlation between chemical shift of H_2_O and water activity a_w_ of hydrated ILs (25 mol%). Region I and II refers to protic ILs with and without H_2_O peak merged with NH peak, respectively, and Region III represented aprotic ILs with linear correlation of chemical shift of H_2_O and a_w_, while the straight line is a guide of the tendency.

Furthermore, chemical shift of H_2_O can be correlated with a_w_, as seen in Figure 5. In the four protic ILs, no clear trend was observed due to the interaction of NH peak with the exchangeable proton of H_2_O. However, based on the anion, the ILs can be classified into the two regions, i.e., EaPn and EoaPn (I) vs. EaMs and TeaMs (II). Region I was coupled with propionate anion showing the merged peak of NH and H_2_O, while region II had single peak of H_2_O (Figure 4). However, comparing EaPn with EoaPn or EaMs with TeaMs, no noticeable difference on the hydrophobicity was observed (Figures 3, 5). This implies that the anion impacts significantly for these two groups of protic ILs.

In terms of the aprotic ILs (region III including ChoMs, ChoPn and ChoDHP), it can be seen that all the H_2_O signals were shifted downfield, suggesting the disruption of the hydrogen bonding network in these ILs. ChoDHP showed the highest shift to downfield, demonstrating the weakest hydrogen bonding with water. This is agreed with the lowest β values and highest a_w_ of ChoDHP. In general, the order of chemical shift of H_2_O also followed the order of a_w_, i.e., ChoDHP, ChoMs and ChoPn (Figure 5). However, ChoMs and ChoPn, have a similar trend with respect to the water peak, while significant differences in β values and a_w_ curves were observed for these two ILs. It is reported that the chemical shift of H_2_O in hydrated aprotic ILs reflected the formation of hydrogen bonding networks between ions and water molecules (Nikawa et al., 2017). Whereas, the relationship between chemical shift of H_2_O and a_w_ in region III showed noticeable deviation on the fitted regression line, it can be suggested that the water peak of aprotic ILs measured by NMR may be not an accurate indicator of hydrophobicity.

### Log P of ILs

Log P has been previously used as a reliable indicator of hydrophobicity (Kaar et al., 2003), however, with respect to the ILs investigated here, Log P via the experimental method could not obtain any useful information. Therefore, the Log P of conjugated base and acid of ILs is considered here. Table 2 shows two Log P values of conjugated base and acid of ILs acquired from two databases. It can be seen that ALog P (a predicted Log P from the database) was generally in good agreement with Log P. However, it is noted that Log P of choline hydroxide was −1.5, which was much higher than its ALog *P*-value (−4.66). In this case, the prediction is probably in the approximation. The Log P of ethanolamine, choline hydroxide and methanesulfonic acid was below −1, indicating that these precursors tend to be hydrophilic. And this low value means 99% of the IL can partition in water and 1% in octanol. For ethylamine and propionic acid with their Log P around 0, they may be slightly hydrophilic. On the contrary, Log P of triethylamine was above 1, indicating that it is hydrophobic. And it is well-acknowledged that triethylamine is water-immiscible. Even though the Log P values for the conjugated base show significant difference i.e., Choline hydroxide (−1.5) and Triethylamine (1.57), the ILs ChoMs and TeaMs both tend to be hydrophilic (Figure 2). This again shows the dominance of the anion determining the hydrophobic nature of the ILs. For the conjugated acids, an inverse relationship exists. For example, propionic acid showed the highest Log P (most hydrophobic), while the propionate-based ILs were the most hydrophilic ILs (Figures 2, 3). This may be due to the spatial conformation change of the anion by the introduction of cations. Unlike the molecular solvents, it is known that ILs network possesses not only hydrogen bonding, but also ionic interactions and Van der Waals forces (MacFarlane et al., 2017). In addition, it is reported that the cation and anion did not contribute equally to the physiochemical properties of ILs (Yang, 2009), and hence the Log P of conjugated base and acid observed in this study do not seem to be relevant for the hydrophobicity of ILs.

**Table 2:** Log *P*-values of the conjugated base and acid of ILs in this study.

**ILs**	**ALogP**	**Log P**	
	**Base/acid**	**Base/acid**	**Cation/anion**
Ethylamine	−0.2	−0.27	−0.27
Triethylamine	1.57	1.26	1.26
Ethanolamine	−1.53	−1.32	−0.98
Choline hydroixde	−1.5	−4.66	−4.66
Propionic acid	0.31	0.48	0.48
Methanesulfonic acid	−2.02	−0.96	−0.96
Phosphoric acid	–	−1.02	−1.02

In relation to the chemical structure of these ILs, propionic acid/propionate has carboxylic group (Figure 1), which is prone to hydrogen bonds with water. Due to the length of alkyl chain, its Log P was slightly high (slightly hydrophobic). When it forms the IL with hydrophilic cations (ethylammonium, ethanolammonium or choline), the hydrophobicity of these ILs significantly decreased. For example, ChoPn was the most hydrophilic ILs among the IL in this study. This may be because the cation forms stronger electrostatic interaction with propionate (and van der Waals dispersion forces), and hence changes the conformation of both cation and anion as well as the hydrogen bonding. Therefore, the hydrogen bonds of IL with water would be different as those of single conjugated base or acid. ChoDHP is the most hydrophilic IL in this study, although it is formed by hydrophilic cation and hydrophilic anion based on their Log P (Table 2). Previous studies have shown that ChoDHP has an extended network of hydrogen bonding particularly though the DHP anions containing two protons (Fujita et al., 2009; Cahill et al., 2010). The proton of DHP anion transports via the reorganization of hydrogen bonds (Rana et al., 2010), and it is possible that this infinite and complicated network of hydrogen bonds hinder the interaction with water, compared with other ILs in this study. Therefore, it would be valuable to compare the measured properties including a_w_ and β to understand hydrophobicity of ILs, rather than just considering Log P or their chemical structure.

## Conclusion

In this work, we evaluated hydrogen bond basicity β and water activity a_w_ as a function of molar concentrations. We suggested these two key measured properties were linearly correlated to each other and related to the hydrophobicity. In addition, the anion dominated these two properties. The order of the hydrophobicity of these ILs can be identified as ChoDHP, mesylate-based ILs, and propionate-based ILs. Then by examining the chemical shift of H_2_O in hydrated ILs from NMR, the hydrophobicity of ILs can be recognized, and we showed that the water peak of hydrated aprotic ILs shifted downfield possibly due to the higher hydrophobicity.

## Author Contributions

QH conducted the experiments, analyzed the data, and wrote the paper. NB and XW supervised the project and critically revised the paper. Both NB and XW were chief investigators of the project. All authors commented on the manuscript and approved the final version to be published.

### Conflict of Interest Statement

The authors declare that the research was conducted in the absence of any commercial or financial relationships that could be construed as a potential conflict of interest.

## Footnotes

^1^*ChemAxon Chemicalize*. Available online at: https://chemicalize.com (accessed May, 11 2018).

^2^*VCCLAB Virtual Computational Chemistry Laboratory*. Available online at: http://www.vcclab.org (accessed May 11, 2018).
